# 2115: Post-traumatic stress disorder associated with Hurricane Katrina predicts cardiovascular disease events among elderly adults

**DOI:** 10.1017/cts.2017.95

**Published:** 2018-05-10

**Authors:** Zachary Lenane, Erin Peacock, Marie Krousel-Wood

**Affiliations:** Tulane University School of Medicine – LA CaTS, New Orleans, LA, USA

## Abstract

OBJECTIVES/SPECIFIC AIMS: Cardiovascular disease (CVD) is the leading cause of death among US adults and its prevalence is increasing, despite efforts to identify, and address risk factors. Post-traumatic stress disorder (PTSD) has been identified as a potential risk factor for CVD, though the results to date have focused on male veterans with combat-related PTSD. To our knowledge, there are no prospective analyses/reports among older community-dwelling adults following Hurricane Katrina. The purpose of this study was to explore the link between PTSD associated with Hurricane Katrina and incident CVD among elderly adults using data from the Cohort Study of Medication Adherence among Older Adults (CoSMO). METHODS/STUDY POPULATION: PTSD associated with Katrina and incident CVD events were assessed among 2075 hypertensive participants age ≥65 who were enrolled in a managed care organization in southeastern Louisiana. Baseline surveys were conducted between August 2006 and September 2007. Baseline surveys were conducted between August 2006 and September 2007. PTSD was assessed using the civilian PTSD CheckList (PCL-17) and 2 cut-off points, ≥37 and ≥44, for primary and secondary analyses, respectively. Participants were followed through February 2011 for a composite CVD outcome of MI, stroke, CHF, or CVD death. Multivariable logistic regression was performed with 13 covariates identified in bivariate analysis: age, sex, race, marital status, education, hypertension knowledge, comorbidities, number of antihypertensive medication classes, dissatisfaction with healthcare, reduced medications due to cost, number of visits to healthcare provider in last year, depression, and coping. RESULTS/ANTICIPATED RESULTS: Participants were 59.8% female and 30.4% black, with a mean age of 75 years. The prevalence of PTSD using the primary and secondary cut points was 6.1% and 4.2%, respectively. In total, 240 (11.5%) participants had a CVD event during a median 3.8 year follow-up. After multivariable adjustment, the odds ratios and 95% confidence intervals (CI) for CVD event for the primary and secondary analyses were 1.90 (95% CI: 1.17, 3.09) and 3.74 (95% CI: 2.05, 6.81), respectively. DISCUSSION/SIGNIFICANCE OF IMPACT: PTSD was associated with an increased risk of incident CVD events among elderly adults. This finding from a prospective cohort study supports earlier reports suggesting PTSD is an independent risk factor for CVD. To our knowledge, this association has not been previously reported among a cohort of elderly community-dwelling adults. This study included hypertensive, elderly, insured participants living in southeastern Louisiana following Hurricane Katrina and may not be generalizable to all people with PTSD. Strengths of this study include its longitudinal design, the identification of incident CVD, the diversity of the study population with respect to gender, race and CV risk, and reduced confounding due to access to care and insurance status. Future research is needed to confirm this finding in other populations and to assess if efforts to minimize the impact of PTSD following disasters reduce CVD risk and premature CVD events among older adults.

